# Tannic Acid Inhibits *Salmonella enterica* Serovar Typhimurium Infection by Targeting the Type III Secretion System

**DOI:** 10.3389/fmicb.2021.784926

**Published:** 2022-01-25

**Authors:** Jingyan Shu, Hongtao Liu, Yang Liu, Xindi Chen, Yu Yu, Qianghua Lv, Jianfeng Wang, Xuming Deng, Zhimin Guo, Jiazhang Qiu

**Affiliations:** ^1^Key Laboratory of Zoonosis, Ministry of Education, College of Veterinary Medicine, Jilin University, Changchun, China; ^2^Department of Clinical Laboratory, The First Hospital of Jilin University, Changchun, China

**Keywords:** *Salmonella* Typhimurium, type III secretion system, tannic acid, anti-virulence agent, anti-infection

## Abstract

*Salmonella enterica* serovar Typhimurium (*S.* Typhimurium) is a zoonotic pathogen that can cause food poisoning and diarrhea in both humans and animals worldwide. The *Salmonella* pathogenicity island (SPI) genes encoded type III secretion system (T3SS) is important for *S.* Typhimurium invasion and replication in host cells. Due to the increasing problem of antibiotic resistance, antibiotic treatment for clinical *Salmonella* infection has gradually been limited. Anti-virulence inhibitors are a promising alternative to antibiotics because they do not easily induce bacterial antibiotic resistance. Here, we systematically evaluated the therapeutic effect of tannic acid (TA) on *Salmonella-*infected mice and elucidated its anti-infection mechanism. TA treatment improved the survival rate of *S.* Typhimurium-infected mice and alleviated cecum pathological lesions. In addition, TA inhibited *S.* Typhimurium invasion to HeLa cells without affecting their growth. Further studies showed that TA could inhibit the expression of *sipA* and *sipB.* This inhibition may be implemented by inhibiting the transcription of key regulatory and structural genes of the T3SS. This study provides an alternative anti-virulence strategy for *Salmonella* infection treatment.

## Introduction

*Salmonella enterica* serovar Typhimurium is a zoonotic foodborne bacterial pathogen that causes human and animal food poisoning worldwide and poses a serious threat to public health and food safety (Kurtz et al., 2017). Currently, antibiotics are extensively used for *S.* Typhimurium infection, but the long-term use of antibiotics inevitably leads to bacterial resistance (Crump et al., 2015). With the increasing antibiotic resistance, the treatment for bacterial infections is becoming nearly ineffective, and an ideal method is urgently needed to fight against infections (Nesterenko et al., 2016).

The anti-virulence strategy is one of the most promising solutions to the problem of antibiotic resistance (Baron, 2010). The *Salmonella* T3SS is a syringe-like precision molecular machine that facilitates bacterial invasion and replication in host cells (Diepold and Armitage, 2015; Finn et al., 2017) and is also an important target for the identification of anti-virulence inhibitors for *Salmonella* infection (Tsou et al., 2016). *Salmonella* invasion of host cells requires the translocation of effector proteins into host cells by the T3SS, such as SipA, SipB, SipC, and SopB, which can promote *Salmonella* invasion of cells by inducing host cell cytoskeletal remodeling (Myeni et al., 2013; Glasgow et al., 2017; Lou et al., 2019). SipA is the key protein of bacteria invading host cells (Zhou et al., 1999; Lou et al., 2019). SipB can also promote *S.* Typhimurium host cell invasion by forming protein complexes with SipC (Myeni et al., 2013). The above information suggests that the T3SS is an ideal target for anti-virulence strategy.

Tannins are water-soluble polyphenols found in almost all plants, and drinking beverages containing tannins can prevent and treat many illnesses (Cowan, 1999). Tannins can inhibit insect growth, help plants fight off herbivorous microbes, protect wood from decay, stimulate phagocytic cells and increase host-mediated tumor activity, and tannins have a wide range of anti-infection functions in humans (Chung et al., 1998; Cowan, 1999). TA is a tannin derivative that has been reported to resist macrophage phagocytosis and improve the survival rate of *S.* Typhimurium-infected mice (Reyes et al., 2017). However, the anti-infection mechanism remains unknown. Here, we found that TA could inhibit *S*. Typhimurium infection by targeting the Type III secretion system. These results indicate that TA is a potential anti-virulence drug for *Salmonella* infection treatment and provide a theoretical reference for the study of anti-*S.* Typhimurium virulence strategy.

## Materials and Methods

### Bacterial Strain, Culture Medium

The *S.* Typhimurium strains SL1344 and SL1344 Δ*invA* were donated by Dr. Xiaoyun Liu from Peking University. SL1344-expressing SipA-beta-lactamase (SipA-TEM) and SL1344 chromosomally expressing Flag-tagged SipB (SipB-3 × Flag) were preserved in our laboratory.

### Animal Experiments

All animal experiments carried out in this study were according to the Institutional Animal Care and Use Committee of Jilin University (number of permit: 2021092035G). A previous report showed that TA improved the survival rate of *S.* Typhimurium-infected mice (Reyes et al., 2017). To comprehensively evaluate the pathological lesions of the intestinal tract and other organs caused by *S.* Typhimurium infection, animal experiments were carried out and described as follows. Healthy BALB/c mice (6–8 weeks old, 20 g) purchased from Changsheng Biotechnology Co., Ltd. (Benxi, China) were kept under 12 h light/dark cycles. The mice were administered streptomycin water (5 g/L) 3 days before oral gavage with *S.* Typhimurium. To determine the survival rate, the infection group mice were subjected to oral administration of SL1344 [100 μL phosphate buffered saline (PBS) with 1 × 10^6^ CFUs], while the control group of mice were given the same volume of bacteria-free 0.9% sodium chloride aqueous solution (SCAS). Two hours before infection, TA (purchased from Chengdu Herb-purify Co., Ltd.) was administered to mice at a dose of 100 mg/kg. Subsequently, TA was administered at an interval of 12 h for 4 days. To assess the bacterial load in organs of infected mice, such as spleen and liver, the aseptically collected tissues were homogenized in sterile SCAS. The homogenate was diluted 10-fold in sterile PBS, plated on 30 μg/mL streptomycin LB agar plates and counted after incubation at 37°C for 12 h. The liver, spleen, and cecum were fixed in 4% paraformaldehyde. To detect the tissue lesions, the fixed tissues were embedded into paraffin followed by hematoxylin-eosin (HE) staining.

### Gentamicin Protection Assay and Immunostaining

A gentamicin protection assay was used to determine *S.* Typhimurium invasion to host cells and was optimized according to a previously described protocol (Galan and Curtiss, 1989) with some modifications. Briefly, HeLa cells were treated with 0.25% trypsin for 3–5 min. The cells were suspended in penicillin-streptomycin-free Dulbecco’s Modified Eagle Medium (DMEM) containing 10% FBS, plated into 24-well plates at a density of 4 × 10^4^ cells per well and cultured at 37°C in a water-jacketed incubator (95% humidity, 5% CO2). Meanwhile, SL1344 WT and Δ*invA* were cultured in LB medium for 12 h. The overnight cultures were inoculated into fresh 0.3 M-NaCl LB with 30 μg/mL streptomycin medium at 1:20, added with TA by gradient concentrations (0, 8, 16, 32 μg/mL), and cultured for another 4 h. The control group was added with the same volume of dimethyl sulfoxide (DMSO). HeLa cells were infected with *S.* Typhimurium at an MOI of 100 for 1.5 h. Extracellular bacteria were washed out by sterile PBS. The 100 μg/mL gentamicin-DMEM was added to each well and incubated for 1 h. HeLa cells were lysed with 500 μL 0.2% (v/v) saponin for 30 min at 37°C after washing with PBS three times. The lysate was serially diluted with sterile water and plated on LB agar plates to determine the colony-forming units (CFUs) of intracellular bacteria. For the immunofluorescence assay, another part of the 24-well HeLa cells was fixed with 4% paraformaldehyde for 20 min at room temperature (RT) (approximately 25°C). Following three washes with PBS, the cells were permeabilized with 0.02% (v/v) Triton X-100 for 5 min and washed with PBS three times. Then, the cells were fixed with 4% goat serum for 20 min. The cells were incubated with rabbit anti-*S.* Typhimurium antibody (ab35156, Abcam) at 1:1000 for 1 h at RT. After three washes with PBS, the cells were incubated with Texas red goat anti-rabbit IgG H&L (T2767, Life Technologies) and 1 μg/mL Hoechst 33342 (C1025, Beyotime) diluted in PBS for 1 h at room temperature. The cells were washed with PBS and observed by fluorescence microscopy (IX83, Olympus).

### *In vitro* Growth Curve of *S.* Typhimurium

Overnight-cultured SL1344 was diluted in 100 mL of 0.3 M NaCl LB at 1:20 and incubated at 37°C until the optical density (OD) at 600 nm (OD_600_) reached 0.3. Then, a gradient concentration of TA was added to the cultures. The OD_600_ was determined using an ultraviolet spectrophotometer (Biophotometer, Eppendorf) at an interval of 1 h for 8 h.

### Determination of Minimum Inhibitory Concentrations

In order to determine the antibacterial efficacy of TA, the MIC assay was performed according to the protocol as follows. The OD_600_ of SL1344 culture was adjusted to 0.1 and diluted 100-fold in fresh LB. The 11-point serial twofold dilutions of TA (2–2048 μg/mL) were prepared in PBS buffer and were added into a 96-well plate. And then, 100 μL SL1344 dilution was added into each well. The 96-well plate with mixture was cultured in 37°C incubator for 16–20 h. The minimum drug concentration without bacterial growth is the MIC of TA for SL1344.

### Determination of the Lactate Dehydrogenase Release

HeLa cells were plated into 96-well plates (2 × 10^4^ cells/well) and cultured for 12 h. Then, the cells were infected (MOI = 50) and incubated with increasing concentrations of TA in DMEM for 5 h. The plates were centrifuged at 1000 *g* for 10 min. The supernatant was collected and detected using the Lactate Dehydrogenase (LDH) cytotoxicity detection kit (11644793001, Roche) on a microplate reader at OD_490__*nm*_.

### The β-Lactamase Assay for the Detection of the Translocation of Type III Secretion System Effector Protein

The 96-well plates were plated with 1.2 × 10^4^ HeLa cells per well. And the plated cells were cultured overnight. The overnight cultures were inoculated into fresh 0.3 M-NaCl LB with 30 μg/mL streptomycin medium at 1:20, added with TA by gradient concentration (0, 4, 8, 16, 32 μg/mL), and cultured for another 4 h. HeLa cells were infected by *S.* Typhimurium at an MOI of 50 for 1.5 h. Plates were centrifuged at room temperature for 5 min at 1,500 rpm and then incubated at 37°C for 1 h. After removing the culture medium, the cells were washed three times with sterile Hank’s balanced salt solution (HBSS) and were covered with 100 μL HBSS containing 20 μL 6 × CCF4/AM (K1095, Life Technologies). The reaction was allowed to proceed for 60 min at RT. The results were observed by fluorescence microscopy (IX83, Olympus).

### Western-Blotting Analysis of the Expression of Type III Secretion System Effector Proteins

The overnight cultures of *S.* Typhimurium expressing SipB-3 × Flag were inoculated into 0.3 M NaCl LB (30 μg/mL streptomycin) with gradient concentrations of TA (0, 8, 16, and 32 μg/mL) and cultured for another 4 h at 37°C. The cell pellet was collected by centrifugation at 12,000 *g* for 2 min. The expression of SipA and SipB proteins was analyzed by Western-Blotting (WB) assay. The primary antibody used for WB analysis of SipA was rabbit anti-SipA IgG (prepared by our laboratory), and anti-Flag mouse IgG (F1804, Sigma) was used for WB analysis of SipB-3 × Flag. The secondary antibodies used for WB were Alexa Fluor-680 goat anti-mouse IgG H&L (ab175775, Abcam) and Alexa Fluor-790 goat anti-rabbit IgG H&L (ab175781, Abcam). Rabbit anti-isocitrate dehydrogenase (ICDH) IgG (ABS2090, Sigma) was probed as a loading control.

### Bacterial Total RNA Extraction and Quantitative Real-Time PCR

The overnight cultures of *S.* Typhimurium expressing SipB-3 × Flag were inoculated into 0.3 M NaCl LB (30 μg/mL streptomycin) with gradient concentrations of TA (0, 8, and 32 μg/mL). They were cultured for another 4 h at 37°C. RNA was extracted using the Bacterial Total RNA Extraction kit (B518625, Sangon Biotech). After that, equal amounts of RNA were reverse transcribed into cDNA using the RevertAid RT reverse transcription Kit (K1691, Thermo Scientific) for analysis. SYBR Green fluorescent dye (KTSM1401, AlpaLife) was used for the Real-Time PCR (RT-PCR) assay. All the samples were repeated three times independently. All the primers used in this study for RT-PCR are shown in Table 1.

**TABLE 1:** Primers for RT-PCR in this study.

Gene name	Primer sequence (5′–3′)	Product size (bp)
*sipA*	CCGGCACCTTGAAATGCAAA	385
	CGAATCCACACGCGAATGAC	
*sipB*	ATGGGATGTATCGGGAAAGT	360
	CTCCATAATCGGGTTTAGCG	
*hilA*	TATCTCCGGGCAGATGATAC	340
	TCTGAGCAAAAGATTCGCAA	
*hilC*	AGCGTATCAAGTCTGAAGCG	147
	ATCATAGCCACACATCGTCG	
*hilD*	TAACGTGACGCTTGAAGAGG	123
	GGTACCGCCATTTTGGTTTG	
*rtsA*	AGGTGGGGAGCATTGAAT	125
	CGTAATTGAAATTTTACCC	
*prgH*	GTTGTGGGCTCGTCAGGTTT	81
	CGCTTATTTTCTTCGTTTTCGT	
*prgI*	AATCTACAAACGCAGGTAA	168
	CTGAATAATGGCAGCATC	
*prgK*	AAAAGGACTGGACCAGGAA	122
	AAATCAGGCTCAGCAACG	
*invG*	GTTTGTTGCGAAAGACGA	165
	CCCAGTTGTAGGGAAAGC	
*gyrB*	TCATTTCCACTACGAAGGCG	111
	CCGAAAAAGACGGTATCGG	

### Statistical Analysis

The experimental data were assessed by unpaired two-tailed *t* tests using GraphPad Prism 8.0 (GraphPad software, La Jolla, CA) and *p* values are indicated as follows: ^***^p < 0.001, ^**^p < 0.01, *p < 0.05.

## Results

### Tannic Acid Can Protect *S.* Typhimurium Infected Mice

**FIGURE 1:**
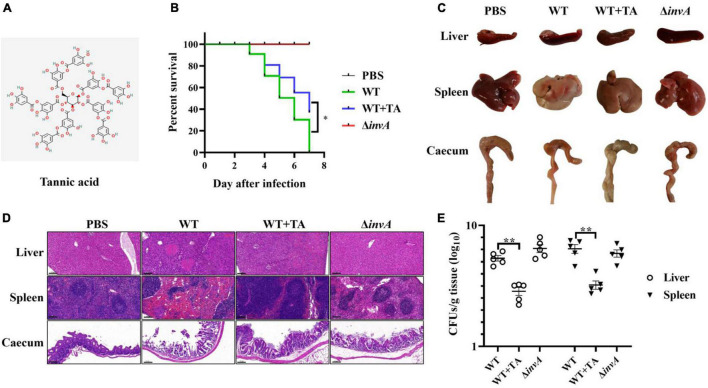
Tannic acid protects mice from *S.* Typhimurium infection. **(A)** The chemical structure of TA. **(B)** TA improved the survival rate of *S.* Typhimurium infected mice (*n* = 10). **(C)** Autopsy analysis of *S*. Typhimurium infected mice. **(D)** Histopathological observations of *S*. Typhimurium infected mice by HE staining. **(E)** The bacterial load in several organs of infected mice on the fifth day post-infection (NS, no significance; *p < 0.05; **p < 0.01, Scale bar, 100 μm).

Tannic acid is a tannin derivative, and the chemical structure of TA is shown in Figure 1A. A previous report showed that TA improved the survival rate of *S.* Typhimurium-infected mice (Reyes et al., 2017). Here, the effect of TA treatment on pathological lesions of the intestinal tract and other organs caused by *S.* Typhimurium infection was comprehensively evaluated. When infected with *S.* Typhimurium, the mice began to die on the third day. The survival rate of the TA-treated group (40%) was significantly increased compared with that of the untreated group at 7 days post-infection (Figure 1B). The autopsy revealed that the cecum showed dehydration and atrophy in the WT-infected mice, while the lesions in the TA-treated group mice were mild. And the PBS and Δ*invA* groups showed no lesions (Figure 1C). Notably, the histopathological analysis showed that the nuclei of hepatocytes in the WT-infected group disappeared and dissolved, which was accompanied by a large number of necrotic and inflammatory foci, neutrophil infiltration and extensive steatosis; there were obvious necrotic foci, central atrophy, and neutrophil infiltration in the spleen; submucosal edema, a large number of exfoliated goblet cells and intestinal villi that appeared in the cecum. However, the TA-treated group and Δ*invA*-infected mice showed mild submucosal edema and goblet cell reduction, which was similar to the PBS control group (Figure 1D). The bacterial load of the liver and spleen was decreased significantly in the TA-treated group compared with the control group (Figure 1E). These data indicated that TA could provide systemic protection from *S.* Typhimurium infection in mice.

### Tannic Acid Inhibited *S.* Typhimurium Invasion Into Host Cells

**FIGURE 2:**
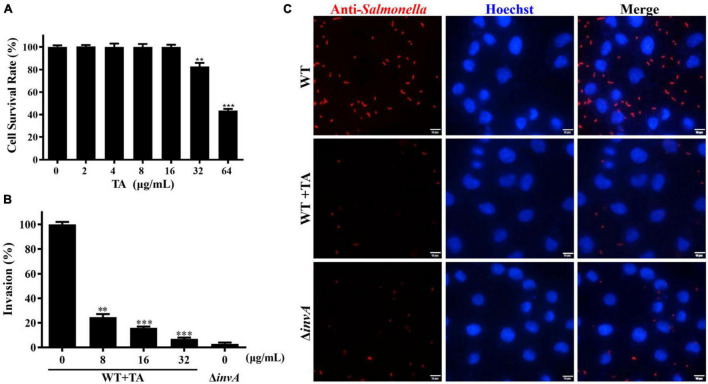
Tannic acid inhibits the invasion of *S.* Typhimurium into HeLa cells. **(A)** The cytotoxity of TA to HeLa cells. **(B)** The effect of TA treatment on the invasion of *S*. Typhimurium into HeLa cells. **(C)** Immunofluorescence analysis for the effect of TA on the invasion of *S*. Typhimurium into HeLa cells (*p < 0.05; **p < 0.01 and ***p < 0.001, Scale bar, 10 μm).

HeLa cells are often used for *Salmonella* infection and the function research of *Salmonella* T3SS effector proteins (Xu et al., 2019; Bourgeois et al., 2021; Walch et al., 2021). TA treatment could provide protection for mice from *S.* Typhimurium infection. The LDH release assay results showed that TA had no cytotoxicity to HeLa cells at concentrations of less than 16 μg/mL (Figure 2A). To explore the mechanism of TA anti-*S.* Typhimurium infection, a gentamicin protection assay was used to analyze the effect of TA-treatment on *S.* Typhimurium invasion into HeLa cells. The results showed that when treated with TA at a concentration of 8 μg/mL, the invasion of HeLa cells by WT was decreased compared with that of the untreated group, while the invasion of Δ*invA* into HeLa cells was hardly observed (Figure 2B). The results of immunofluorescence analysis demonstrated that many bacteria gathered around the cells in the WT-infected group. TA treatment inhibited this gathering phenomenon (Figure 2C). These results indicated that TA could effectively inhibit the invasion of HeLa cells by *S.* Typhimurium.

### Tannic Acid Inhibited the Translocation of SipA-TEM Expressed in *S*. Typhimurium

**FIGURE 3:**
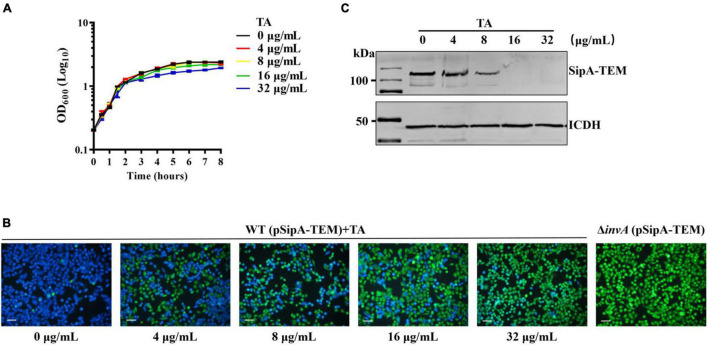
Tannic acid inhibits translocation of SipA-TEM expressed in *S.* Typhimurium. **(A)** The effect of TA on *S.* Typhimurium growth. **(B)** The effect of TA treatment on the translocation of SipA-TEM expressed in *S.* Typhimurium into HeLa cells (Scale bar, 100 μm). **(C)** The effect of TA treatment on the expression of SipA-TEM in *S*. Typhimurium.

Both the *in vivo* and *in vitro* assays showed that TA could protect the host from *S.* Typhimurium infection. The underlying mechanism of TA remains unclear. Thus, the effect of TA treatment on the translocation of *S.* Typhimurium T3SS was detected by a β-lactamase reporting system. The minimum inhibitory concentrations (MICs) of TA to *S.* Typhimurium was higher than 2048 μg/mL. TA had no effect on the growth of *S.* Typhimurium at concentrations of less than 32 μg/mL (Figure 3A). SipA is the key protein for bacterial invasion to host cells (Zhou et al., 1999; Lou et al., 2019). When HeLa cells were infected with *S.* Typhimurium expressing SipA-TEM, obvious translocation of SipA was observed. SipA-TEM expressed in Δ*invA* failed to translocate SipA. When incubated with TA, the translocation of SipA was inhibited. The inhibitory effect of TA on the translocation was enhanced in a dose-dependent manner (Figure 3B). Further WB results showed that TA inhibited the expression of SipA-TEM in *S.* Typhimurium (Figure 3C). Thus, TA could inhibit bacterial invasion of host cells by inhibiting the expression of SipA-TEM.

### Tannic Acid Inhibited the Expression of Type III Secretion System Effector Proteins

**FIGURE 4:**
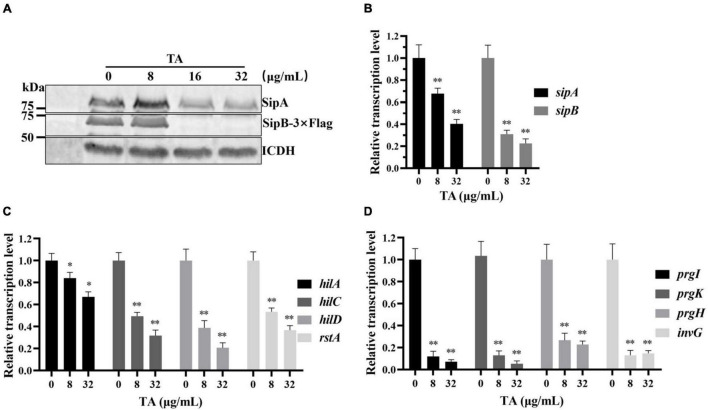
Tannic acid inhibits the expression of T3SS effector proteins by inhibiting the transcription of key SPI-1 genes. **(A)** The effect of TA on chromosomally expression of *sipA* and *sipB* genes by WB analysis. **(B)** The effect of TA on transcription of *sipA* and *sipB* genes by RT-PCR. **(C)** The effect of TA on transcription of regulatory genes by RT-PCR. **(D)** The effect of TA on transcription of structural genes of T3SS by RT-PCR (*p < 0.05, ^**^p < 0.01).

To clarify the mechanism by which TA inhibits the translocation of SipA, WB analysis was used to detect the expression of endogenous SipA. The results indicated that TA-treatment inhibited endogenous SipA expression and the inhibitory effect was enhanced in a dose-dependent manner (Figure 4A). SipB can also promote *S.* Typhimurium invasion of host cells (Myeni et al., 2013). Endogenous SipB-3 × Flag was also detected by WB, and similar inhibition of SipB was observed (Figure 4A).

### Tannic Acid Inhibited the Transcription of Key Effector Genes and Regulatory and Structural Genes of the Type III Secretion System

Real-Time PCR was used to evaluate the effect of TA on the transcription levels of the *sipA* and *sipB* genes. TA inhibited the expression of T3SS effector proteins. The transcription levels of the *sipA* and *sipB* genes were significantly decreased in the TA-treated group compared with the other groups (Figure 4B). Previous reports showed that HilC, HilD, and RstA promoted the expression of *hilA* by binding to the *hilA* promoter region (Schechter and Lee, 2001; Olekhnovich and Kadner, 2002). InvG, PrgH, PrgK, and PrgI are structural components of the *S.* Typhimurium T3SS. HilA is a key upregulator of the expression of T3SS component genes and T3SS effector genes (Brown et al., 2014). The RT-PCR results showed that the transcription levels of *hilC, hilD, hilA, rstA* (Figure 4C) *and invG*, *prgH, prgK*, *prgI* were significantly inhibited by TA in a dose-dependent manner (Figure 4D). In short, TA inhibits the expression of T3SS effector proteins and may be implemented by inhibiting the transcription of key regulatory and structural genes of the T3SS.

## Discussion

Antibiotic resistance in *Salmonella* is a growing problem (Bhutta, 2006; Koirala et al., 2012; Parry et al., 2013). The anti-virulence strategy is an ideal way to solve increasing antibiotic resistance problems (Nesterenko et al., 2016), which only targets bacterial virulence without affecting bacterial growth. Anti-virulence inhibitors do not put serious strain on bacterial survival, which greatly reduces the likelihood of inducing drug resistance (Rasko and Sperandio, 2010). It is becoming a trend to develop small molecular compounds targeting bacterial virulence factors (Duncan et al., 2012). For example, baicalin can covalently inactivate T3SS effector proteins (Tsou et al., 2016). Salicylhydrazide has extensive inhibitory effects on the activity of the T3SS of several gram-negative pathogens, including *S.* Typhimurium, *Pseudomonas aeruginosa*, and *Chlamydia* (Negrea et al., 2007; Ur-Rehman et al., 2012; Anantharajah et al., 2017).

Tannic acid can improve the survival rate of *Salmonella-*infected mice (Reyes et al., 2017). This study clarified the anti-infection mechanism of TA. TA can inhibit the transcription of the *sipA* and *sipB* genes by suppressing the *hilD*-*hilC*-*rstA*-*hilA* regulatory network, consequently reducing the expression of invasion-related molecules such as the downstream effector proteins SipA and SipB and ultimately inhibit the invasion of *S.* Typhimurium. In addition, this study analyzed the pathological damage of tissues in *Salmonella*-infected mice treated with TA and showed that TA significantly alleviated the intestinal damage caused by *S.* Typhimurium infection. These results enrich the evidence of the TA therapeutic effect on *S.* Typhimurium infection.

It is well known that there may be many mechanisms for the inhibition of T3SS by small molecular compounds. First, small molecular compounds may directly inhibit the transcription of upstream related regulatory genes. Second, the compounds may directly interact with one or more main substrates of the T3SS to make them inactive, thus damaging the integrity of the T3SS. Third, the compounds may affect the expression and secretion of multiple effector proteins (Myeni et al., 2013; Owen et al., 2014; Jennewein et al., 2015). This study found that TA not only reduced the expression of SipA and SipB but also inhibited the transcription of *invG, prgI, prgH*, and *prgK*, all of which encode proteins that are structural components of the T3SS. Therefore, TA inhibited *S.* Typhimurium invasion of host cells, possibly by inhibiting the translocated function of the T3SS. This is one of the main directions of our follow-up research. In addition, the results of this study demonstrated that TA could improve the survival rate of *S.* Typhimurium-infected mice, but its half-life and other important pharmacokinetic indices in mice were not analyzed. Therefore, we will also elucidate in detail the pharmacokinetic parameters of TA in mice in the future.

## Conclusion

Tannic acid inhibited *S.* Typhimurium infection by targeting the Type III secretion system, significantly reduced mortality and effectively alleviated tissue damage in *S.* Typhimurium-infected mice. This provides a potential anti-virulence drug for *Salmonella* infection treatment.

## Data Availability Statement

The original contributions presented in the study are included in the article/supplementary material, further inquiries can be directed to the corresponding author/s.

## Ethics Statement

The animal study was reviewed and approved by the Institutional Animal Care and Use Committee of Jilin University (number of permit: 2021092035G).

## Author Contributions

JQ and ZG designed this study. JS, YL, and XC completed the animal experiments. YY and HL completed the *in vitro* assays. HL, QL, and JS analyzed the data. HL and JS wrote the manuscript. JQ, XD, and JW revised the manuscript. All authors contributed to the article and approved the submitted version.

## Conflict of Interest

The authors declare that the research was conducted in the absence of any commercial or financial relationships that could be construed as a potential conflict of interest.

## Publisher’s Note

All claims expressed in this article are solely those of the authors and do not necessarily represent those of their affiliated organizations, or those of the publisher, the editors and the reviewers. Any product that may be evaluated in this article, or claim that may be made by its manufacturer, is not guaranteed or endorsed by the publisher.
